# NI-RADS in posttreatment head and neck cancer surveillance: a framework for standardized imaging with clinical impact

**DOI:** 10.1186/s40644-026-00989-y

**Published:** 2026-01-10

**Authors:** David A. Zander, Ashley H. Aiken, Yuh-Shin Chang, Fabian Elsholtz, Ryan Hughes, Amy F. Juliano, Kim O. Learned, Ashok Srinivasan, Sara Strauss, Jaime Wicks, Paul M. Bunch

**Affiliations:** 1 Department of Radiology, University of Colorado School of Medicine, Aurora, CO United States of America; 2 Department of Radiology and Imaging Sciences, Emory University School of Medicine, Atlanta, GA United States of America; 3 Department of Radiology, Massachusetts Eye and Ear, Harvard Medical School, Boston, MA United States of America; 4 Department of Radiology, Charité–University Medical Center, Berlin, Germany; 5 Department of Radiation Oncology, Wake Forest University School of Medicine, Winston-Salem, NC United States of America; 6 Department of Radiology, Massachusetts Eye and Ear, Harvard Medical School, Boston, MA United States of America; 7 Department of Neuroradiology, Division of Diagnostic Imaging, The University of Texas MD Anderson Cancer Center, Houston, TX United States of America; 8 Department of Radiology, University of Michigan, Ann Arbor, Michigan United States of America; 9 Department of Radiology, Weill Cornell Medical College, New York, NY United States of America; 10 Department of Radiology, Wake Forest University School of Medicine, Winston-Salem, NC United States of America

## Abstract

The Neck Imaging Reporting and Data System (NI-RADS), developed through the American College of Radiology (ACR), provides a standardized framework for interpreting and managing posttreatment imaging in head and neck cancer. Building upon the success of NI-RADS PET/CT, the recently released NI-RADS MRI version 2025 represents a major advancement, introducing modality-specific descriptors and management recommendations tailored to MRI. This review summarizes the history and development of NI-RADS, highlighting both the validated PET/CT framework and the subsequent MRI update. At its core, NI-RADS offers a standardized lexicon for post-treatment findings, a structured reporting format that stratifies risk of disease recurrence, and linked management recommendations. Surveillance imaging is an essential component of post-treatment head and neck cancer care. Evidence strongly supports early imaging surveillance within six months of definitive therapy, whereas the benefits of long-term imaging surveillance remain under-investigated. With the goal of improving patient outcomes, NI-RADS provides a consistent, risk-adaptable framework that supports both clinical decision-making and standardized data collection for future outcomes research. NI-RADS will continue to evolve with advances in oncologic management and imaging technology. Future updates may incorporate ultrasound, advanced imaging techniques, and circulating tumor biomarkers. As surveillance strategies in head and neck cancer advance, NI-RADS is well positioned to serve as the foundation for personalized, risk-based imaging surveillance.

## Background

The Neck Imaging Reporting and Data System (NI-RADS) was proposed through the American College of Radiology (ACR) to bring uniformity, reproducibility, and risk stratification to the challenging task of interpreting surveillance imaging studies performed after definitive treatment for head and neck cancer. This article offers an updated overview of NI-RADS, with a focus on the newly released 2025 MRI version. We cover the diagnostic rationale, evidence base, and structure of NI-RADS, summarize the system’s performance, highlight the value of NI-RADS as a surveillance paradigm, and explore future directions.

## History

The ACR introduced its first Reporting and Data System (RADS) in 1993 with the Breast Imaging Reporting and Data System (BI-RADS). Developed to address lack of standardization and uniformity in screening mammography, BI-RADS established a lexicon of imaging descriptors, a structured reporting format incorporating risk assessment, and management recommendations linked to each risk category. This framework for data collection and auditing also enabled quality improvement and research. The success of BI-RADS led to the development of additional RADS, such as Lung-RADS for lung cancer screening, LI-RADS for informing the likelihood of hepatocellular carcinoma in at-risk patients, and TI-RADS for thyroid nodule risk-stratification.

NI-RADS follows the same structure with standardized lexicon, risk stratification, and linked management recommendations but focuses on posttreatment surveillance of head and neck cancer instead of primary screening. It assesses both the primary site and cervical nodes, aiming to improve communication, consistency, and data-driven quality improvement.

Originally described in 2016 [1] and published as an ACR White Paper in 2018 for computed tomography (CT) and 18 F-fluorodeoxyglucose (FDG) positron emission tomography (PET)/CT [2], NI-RADS was developed by experts in head and neck imaging who prioritized multidisciplinary collaboration [3]. This group sought to standardize complex post-treatment imaging findings into a shared lexicon of independent imaging features categorized by relative risk of recurrent disease and linked to further management steps, such as modification of the surveillance regimen or biopsy. While the original CT and FDG PET/CT-based system could theoretically be modified to apply to MRI, no guidance specific to the interpretation of surveillance MRI was initially provided.

**Fig. 1:**
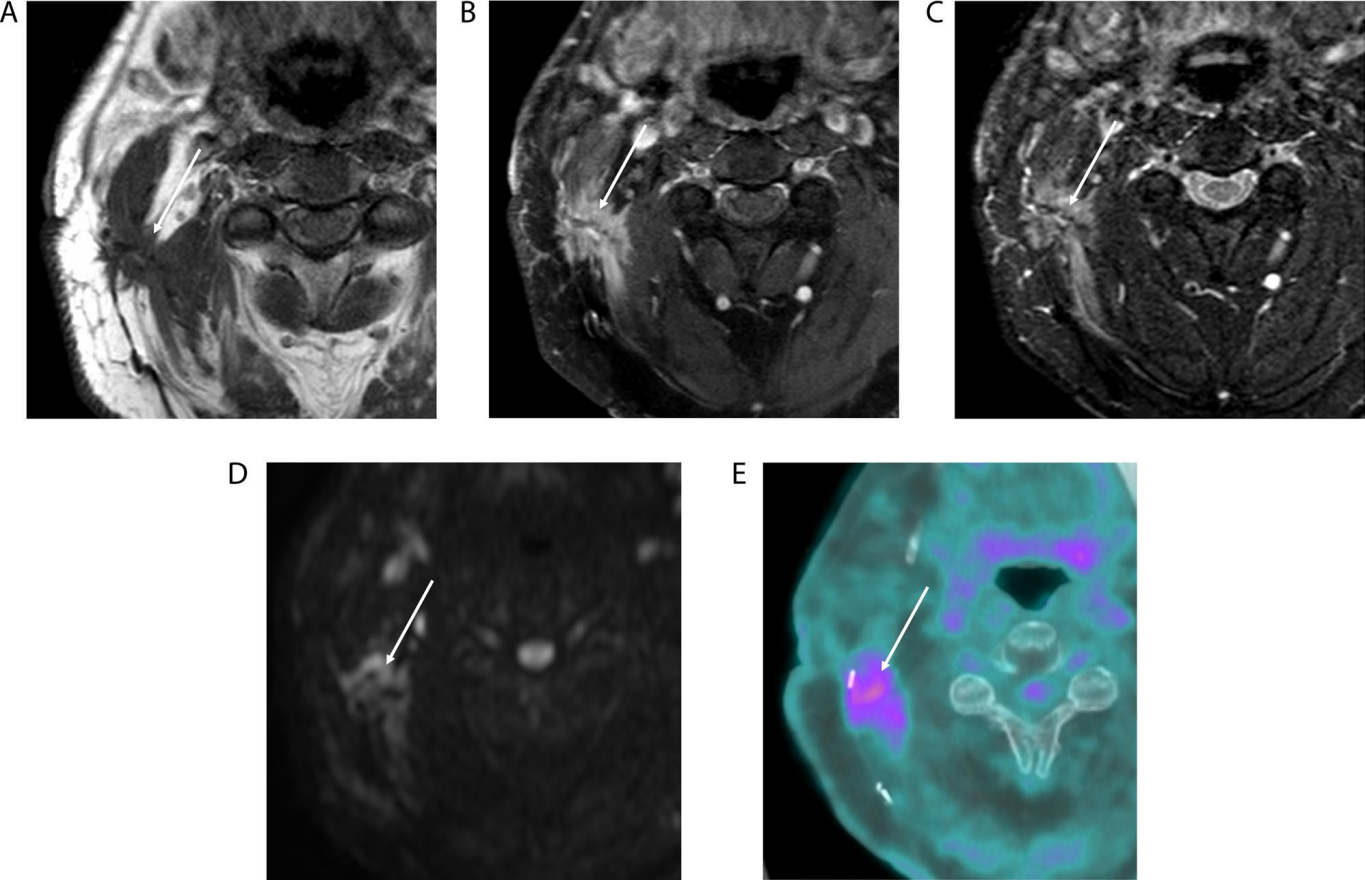
Recurrent HNSCCA at the deep margin of a free flap reconstruction, NI-RADS MRI primary category 3. Axial T1-weighted (**A**), Axial T1-weighted fat-suppressed contrast-enhanced(**B**), Axial T2-weighted fat-suppressed (**C**), and Axial diffusion-weighted MR (**D**) images demonstrate the utility of T2-weighted and diffusion-weighted images to distinguish recurrent tumor (arrows) from surrounding post treatment changes, with intermediate T2 signal intensity and low diffusivity, respectively. This is confirmed by axial fused FDG PET/CT (**E**) with corresponding intense FDG uptake

Recognizing the advantages of MRI in surveillance imaging in certain scenarios - particularly for tumors of the skull base, nasopharynx, salivary glands, and sinonasal region - the ACR NI-RADS Committee recently released a consensus-based version of NI-RADS dedicated to MRI: ACR NI-RADS MRI version 2025 [4]. This article is the most significant update to NI-RADS since its inception and highlights features on T2-weighted and diffusion-weighted imaging (DWI) that show utility for distinguishing recurrent head and neck cancer from posttreatment change [5–7] (Fig. 1). The new version includes specific criteria and management algorithms for MRI, which mirror the earlier NI-RADS PET/CT, along with guidance for evaluating perineural spread and other findings for which MRI is superior to CT or PET. Although there is no standardized MRI protocol for head and neck cancer surveillance, NI-RADS MRI 2025 echoes previous expert recommendations for non-contrast T1-weighted images without fat suppression, T2-weighted images, and contrast-enhanced T1-weighted images with fat suppression, each in at least two planes [8]. A diffusion-weighted sequence is also advised. The Dixon fat suppression technique is suggested to minimize artifacts, and targeted high-resolution imaging of the primary site is appropriate, particularly when other modalities such as CT and/or ultrasound are used to assess the neck.

## Surveillance imaging: NI-RADS as a framework

The primary goal of surveillance imaging after definitive treatment for head and neck cancer is to detect recurrent disease when it can be most effectively treated with salvage therapy. Regimented clinical evaluation with history and physical examination remains the most effective tool for detecting recurrence and is the foundation for surveillance [9]. Most recurrences are detected when patient symptoms lead to diagnosis [10, 11]. However, many symptomatic recurrences are at an advanced stage when salvage therapy may no longer be an option. Surveillance imaging aims to detect disease earlier - before symptoms arise and when salvage therapy may still be possible – with the potential for improved outcomes.

There is strong evidence to support the use of imaging, especially PET/CT, within the first 6 months after definitive therapy, defined as the initial response assessment period [10, 12]. PET/CT performed at 12 weeks shows high accuracy for detecting residual or recurrent disease, with pooled sensitivity and specificity of 86.2% and 82.3% for primary sites and with greater specificity for distant metastases [12].

The benefit of long-term surveillance imaging, defined as greater than 6 months following a complete response, is unclear due to limited, heterogenous retrospective data and insufficient evidence [13–15]. While National Comprehensive Cancer Network (NCCN) guidelines Version 4.2025 recommend imaging within 6 months of treatment completion, they also state that routine imaging beyond a negative 3-month PET/CT offers no proven benefit in asymptomatic patients [10]. Despite this, long-term imaging surveillance is commonly practiced in clinical settings [16]. Approximately 95% of asymptomatic head and neck squamous cell carcinoma (HNSCC) recurrences are detected within 2 years of finishing treatment [17], supporting surveillance during that period [18]. Late recurrence of HNSCC does occur, and other tumor types, such as adenoid cystic carcinoma are known for delayed relapse, necessitating extended follow-up [19]. Imaging detection of metachronous primary cancers may improve survival benefit [15, 20]. The NCCN guidelines recommend that annual low-dose chest CT be considered for patients with head and neck cancer and a history of heavy smoking to screen for pulmonary metastases or second primary lung cancer [10]. Recent Appropriate Use guidelines published by the American Radium Society emphasize the need for personalized surveillance based on individual risk profiles [21].

Furthermore, whereas the value of long-term surveillance imaging in early-stage disease is unclear [13, 14], evidence suggests that patients diagnosed with advanced-stage disease may benefit from continued imaging surveillance [20, 22].

While no clear survival benefit has been demonstrated when long-term surveillance imaging is performed without a structured regimen [13], some studies show that structured, scheduled imaging, especially with iodinated contrast-enhanced PET/CT, can improve outcomes [15, 20]. These heterogeneous and limited data, along with associated challenges of personalized treatment, contribute to the current lack of strong consensus guidelines regarding the timing, duration, and preferred modality of surveillance imaging—leading many institutions to develop their own protocols.

In contrast, NI-RADS is rooted in evidence for short-term imaging surveillance and is uniquely positioned as a structured and personalizable risk-adaptable tool for long-term imaging surveillance, filling a critical gap in national imaging surveillance guidelines and providing a framework for future outcomes research.

## NI-RADS categories and diagnostic performance

When interpreting a posttreatment imaging study, evaluation for recurrent disease is directed both at the primary site and regional lymph nodes, with NI-RADS scores assigned to each. As some institutional MR imaging protocols may be tailored to the primary site and therefore exclude the entirety of the neck, the NI-RADS MRI neck score may be qualified as “X – not assessed.”

**Fig. 2:**
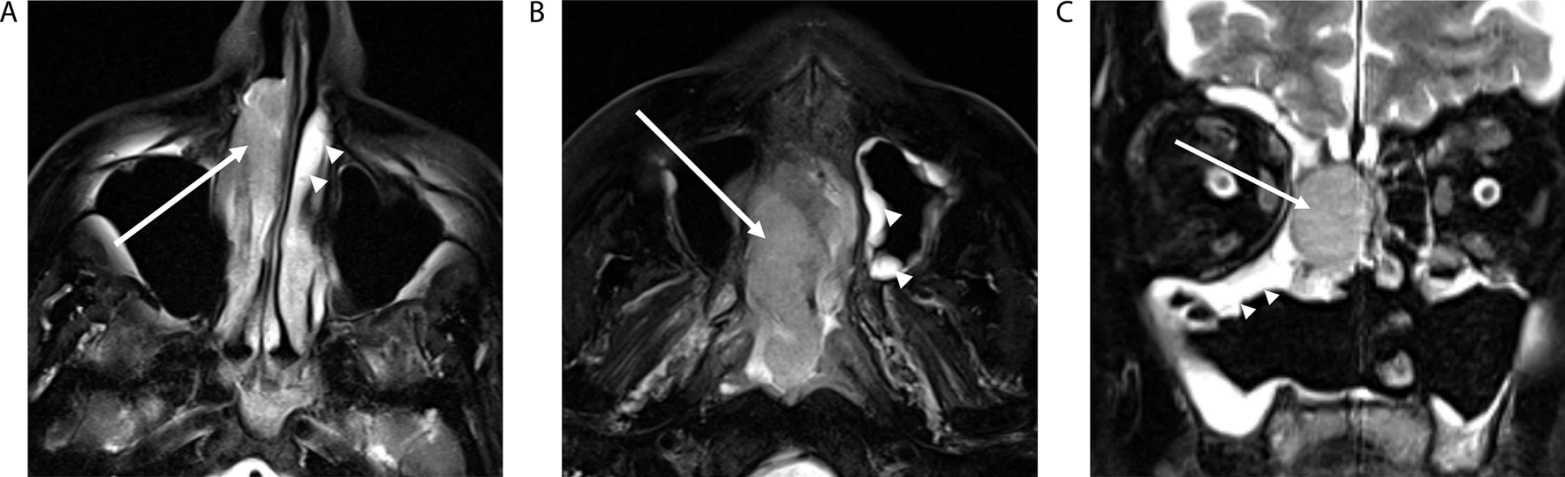
Axial T2-weighted fat-suppressed (**A** and **B**) and coronal T2-weighted fat-suppressed (**C**) MR images in a patient with multiple-recurrent sinonasal mucosal melanoma with identical intermediate T2 signal intensity of the tumor (arrows) compared to the T2 hyperintense signal in the normal mucosa (arrowheads) at initial staging (**A**), NI-RADS MRI primary category 3 first recurrence (**B**), and NI-RADS primary category 3 second recurrence (**C**)

NI-RADS 0 is used when known relevant prior imaging is not available and would impact surveillance interpretation. Because recurrent tumor often exhibits imaging features similar to those of the original malignancy (Fig. 2), comparison to pre-treatment imaging is essential when interpreting posttreatment surveillance imaging [23]. Comparison to prior post-treatment imaging is also prudent because new findings associated with the primary site or neck lymph nodes should generally be viewed with higher suspicion than findings that are unchanged in comparison to multiple prior examinations.

**Fig. 3:**
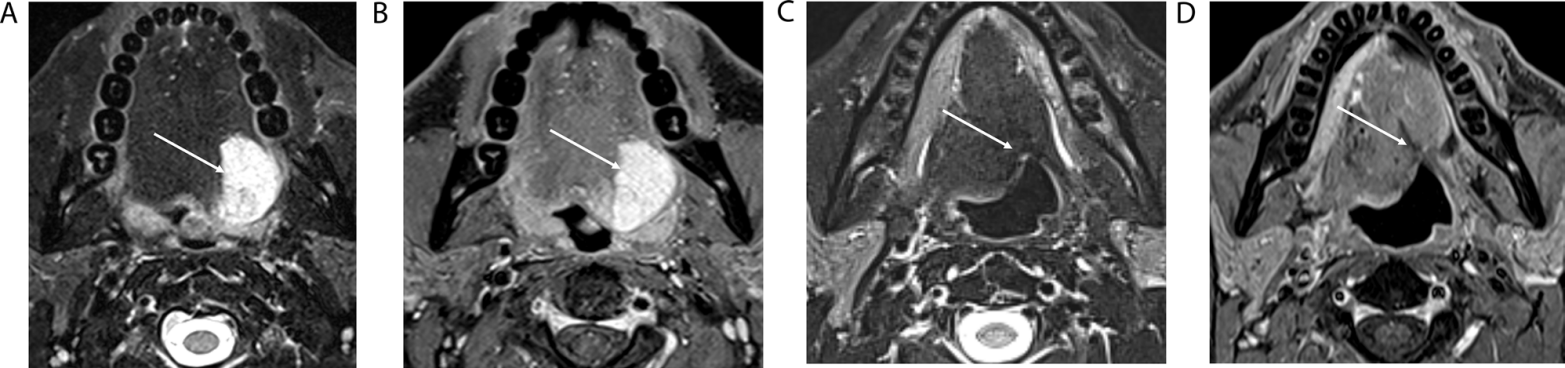
Axial T2-weighted fat-suppressed (**A**) and axial T1-weighted fat-suppressed post contrast (**B**) MR images of an adenoid cystic carcinoma (arrows) of the left base of tongue at initial staging. Axial T2-weighted fat-suppressed (**C**) and axial T1-weighted fat-suppressed post contrast (**D**) MR images of the same patient following definitive treatment with resection and adjuvant radiation therapy with features of NI-RADS MRI primary category 1 including non-mass-like distortion of soft tissues with low T1 and low T2 signal intensity suggesting scar/fibrosis (arrows)

NI-RADS 1 indicates no evidence of recurrence and reflects either normal posttreatment findings or expected changes (Fig. 3). It has a high NPV across modalities. For CT and PET/CT, recurrence rates are 4.2% (95% CI, 2.6%-6.7%) for primary sites and 3.5% (95% CI, 2.1%-5.8%) for nodal disease [24], while recent evaluations of NI-RADS PET/CT applied to MRI report similar high NPV [6]. This category supports routine surveillance with no immediate intervention required.

NI-RADS 2 indicates low suspicion for recurrence. Primary sites are further subdivided into 2a for mucosal abnormalities and 2b for deep findings. This category has potential for greater variability of interpretation because of overlapping appearances of posttreatment tissue change, inflammation, and fibrosis. Recurrence rates for CT and PET/CT are 29.0% (95% CI, 21.2%-38.2%) for primary sites and 14.3% (95% CI, 6.1%-30.0%) for nodal disease [24]. Recommendations include direct endoscopic inspection for primary site mucosal findings or short-term follow-up imaging for primary site findings located deeper in the neck or associated with the cervical lymph nodes. Because FDG PET is relatively insensitive to perineural tumor spread [25], MRI is preferred over PET/CT when there is clinical or imaging concern for perineural recurrence [4].

NI-RADS 3 indicates high suspicion for recurrence. Recurrence rates for CT and PET/CT are 74.4% (95% CI 59.2%-85.4%) for primary sites and 73.3% (95% CI, 63.0%-81.5%) for nodal disease [24]. Early MRI studies using DWI (ADC_mean_ threshold of 1.3 × 10 ^− 3^ mm^2^/s) and intermediate T2 signal show PPV over 80% for both primary and nodal assessments [5, 6]. The linked recommendation is biopsy.

NI-RADS 4 indicates known or proven recurrence. Typically, biopsy has already been performed or has been deemed unnecessary by the managing head and neck cancer multidisciplinary team. Linked management is directed by the clinical team.

### Cessation of imaging surveillance

There is evidence to support discontinuation of surveillance imaging after two consecutive negative FDG PET/CTs, spaced six months apart, with reported negative predictive value of 98% [26]. This evidence is incorporated into NI-RADS with recommendation for cessation of imaging surveillance when PET and contrast-enhanced CT are negative both at 8–12 weeks post-treatment and six months later [2, 4]. Most recurrences of HNSCC occur within two years of completing definitive therapy [17]. Otherwise, there are limited data to define the optimal duration for long-term imaging surveillance. For patients undergoing contrast-enhanced CT alone, continued annual imaging may be appropriate, with or without chest CT. Where clinically indicated, MRI can be used in place of CT. Additionally, the NI-RADS framework is applicable to imaging prompted by clinical concern for recurrence even after completion of routine asymptomatic surveillance.

## Evolving concepts and future direction

### NI-RADS validation

The NI-RADS MRI 2025 release is expected to drive new validation efforts to determine if MRI categories follow similar recurrence rates to those reported for NI-RADS PET/CT. PET/CT-based NI-RADS has shown growing success in long-term surveillance. One study of 255 cases with NI-RADS 2 and 3 found that imaging-detected recurrence preceded clinical exam findings in over one-third of cases and in 81% of asymptomatic recurrences beyond six months [27]. Emerging data suggest that structured and personalized long-term imaging surveillance improves patient care; however, further outcomes-based research is required to determine whether NI-RADS adoption confers such benefits. Assuming positive benefits, further investigation could explore the ideal frequency and duration of long-term imaging surveillance at a risk-adjusted level [28].

### Tumor-tissue modified viral HPV DNA (TTMV-HPV DNA)

A promising development in head and neck cancer surveillance is the use of TTMV-HPV DNA, particularly in HPV-associated oropharyngeal cancers, as an increased plasma level may indicate the presence of recurrence before detectable by imaging [29, 30]. The presence of plasma TTMV-HPV DNA after definitive therapy is highly specific and sensitive for recurrence [31]. In high-risk patients or in the setting of indeterminate clinical or imaging findings, TTMV-HPV DNA can help guide clinical decision making [28, 32]. All panelists on the ARS Appropriate Use Committee agreed that biomarker surveillance with TTMV-HPV DNA “may be” or is “usually” appropriate for patients with a pre-treatment positive result [21]. While not yet incorporated into NI-RADS, emerging evidence may justify the integration of TTMV-HPV DNA into future iterations to personalize surveillance and potentially improve imaging specificity.

### Advances in MRI

NI-RADS MRI 2025 represents a foundation for future development. Advanced MRI techniques like dynamic contrast-enhanced imaging, arterial spin labeling, and perfusion metrics may increase accuracy of surveillance [8]. Radiomics and machine learning could improve lesion classification and prediction of recurrence [33]. PET/MRI could add value by combining the metabolic and morphologic evaluations [34].

### Ultrasound in NI-RADS surveillance

Ultrasound (US) has been extensively studied for detecting cervical nodal metastases in both initial staging and surveillance settings. A meta-analysis found that US and US-guided fine needle aspiration cytology were more accurate than CT and MRI for detecting cervical lymph node metastases [35]. Individual sonographic features, such as loss of hilar architecture, round shape, cystic change, and microcalcifications, were independently predictive of malignancy, and the presence of more than 1 feature further increased risk of malignancy [36].

A recent retrospective cohort study reported that in NI-RADS node 2 cases, ultrasound had over 95% accuracy for recurrence in the cohort [37]. In NI-RADS node 3 cases, where the linked recommendation is biopsy, a normal US could downgrade the node to NI-RADS node 1 and thereby potentially avoid the invasiveness of a biopsy [37].

### Immunotherapy

NCCN guidelines support immunotherapy for patients with unresectable, recurrent, and/or metastatic HNSCC [10]. As immunotherapy alone is not considered definitive therapy, associated imaging does not fall under NI-RADS surveillance paradigms. However, emerging data show improved overall survival with Pembrolizumab in the neoadjuvant and adjuvant settings for locally advanced resectable disease [38]. Similarly, Cemiplimab has shown promise as a first-line therapy for locally advanced cutaneous HNSCC with many patients achieving complete pathologic response [39–41]. Radiologists can anticipate seeing immunotherapy-related changes when used as adjuvant therapy, and there may be an emerging role for shifting patients to a surveillance imaging paradigm when first-line immunotherapy is successful. Future iterations of NI-RADS could potentially add descriptors and risk-stratification for immunotherapy-related imaging findings [42].

### Implementation and integration

Implementation of NI-RADS is expected to benefit patient care but requires multidisciplinary team buy-in and radiologist training. Structured surveillance report templates improve clarity, support multidisciplinary tumor board discussions, and reduce interpretive variability [1, 23]. Overall, adoption of NI-RADS has been favorably viewed by both referring clinicians and interpreting radiologists [43]. Recognizing that many institutions have existing protocols for imaging surveillance, local implementation of NI-RADS can be adapted to best meet the needs of the multidisciplinary head and neck cancer team. The system’s flexibility and evidence-informed design support integration into a variety of practice settings.

## Conclusion

NI-RADS is an evidence-based imaging paradigm designed to support head and neck cancer surveillance through structured, concise, reproducible and clear reporting. Its iterative development ensures responsiveness to the evolving needs of radiologists and head and neck oncology practitioners. For example, NI-RADS MRI version 2025 incorporates the latest evidence in MRI-based head and neck cancer surveillance and builds upon the strong and well-established foundation of NI-RADS PET/CT. The reproducible NI-RADS framework enables precise categorization of posttreatment findings with accompanying management guidance for both short-term and long-term imaging surveillance. NI-RADS also facilitates quality improvement and future research. As surveillance strategies continue to evolve, NI-RADS offers a framework for delivering consistent, personalized, risk-based imaging follow-up in head and neck oncology.

## Data Availability

No datasets were generated or analysed during the current study.

## Abbreviations

ACR: American College of Radiology
CT: Computed tomography
DWI: Diffusion-weighted imaging
FDG: 18 F-fluorodeoxyglucose
HNSCC: Head and neck squamous cell carcinoma
NI-RADS: Neck Imaging Reporting and Data System
PET: Positron emission tomography
RADS: Reporting and Data System
TTMV-HPV DNA: Tumor-tissue modified viral HPV DNA
